# Mechanistic Insights of Anti-Immune Evasion by Nobiletin through Regulating miR-197/STAT3/PD-L1 Signaling in Non-Small Cell Lung Cancer (NSCLC) Cells

**DOI:** 10.3390/ijms22189843

**Published:** 2021-09-11

**Authors:** Nipin Sp, Dong Young Kang, Jin-Moo Lee, Kyoung-Jin Jang

**Affiliations:** 1 Department of Pathology, School of Medicine, Institute of Biomedical Science and Technology, Konkuk University, Chungju 27478, Korea; nipinsp@konkuk.ac.kr (N.S.); kdy6459@kku.ac.kr (D.Y.K.); 2 Pharmacological Research Division, National Institute of Food and Drug Safety Evaluation, Osong Health Technology Administration Complex, Cheongju-si 28159, Korea; elzem@korea.kr

**Keywords:** nobiletin, NSCLC, PD-L1, EGFR, JAK2/STAT3, miR-197, anti-PD-1 mAb

## Abstract

Tumor immune escape is a common process in the tumorigenesis of non-small cell lung cancer (NSCLC) cells where programmed death ligand-1 (PD-L1) expression, playing a vital role in immunosuppression activity. Additionally, epidermal growth factor receptor (EGFR) phosphorylation activates Janus kinase-2 (JAK2) and signal transduction, thus activating transcription 3 (STAT3) to results in the regulation of PD-L1 expression. Chemotherapy with commercially available drugs against NSCLC has struggled in the prospect of adverse effects. Nobiletin is a natural flavonoid isolated from the citrus peel that exhibits anti-cancer activity. Here, we demonstrated the role of nobiletin in evasion of immunosuppression in NSCLC cells by Western blotting and real-time polymerase chain reaction methods for molecular signaling analysis supported by gene silencing and specific inhibitors. From the results, we found that nobiletin inhibited PD-L1 expression through EGFR/JAK2/STAT3 signaling. We also demonstrated that nobiletin exhibited p53-independent PD-L1 suppression, and that miR-197 regulates the expression of STAT3 and PD-L1, thereby enhancing anti-tumor immunity. Further, we evaluated the combination ability of nobiletin with an anti-PD-1 monoclonal antibody in NSCLC co-culture with peripheral blood mononuclear cells. Similarly, we found that nobiletin assisted the induction of PD-1/PD-L1 blockade, which is a key factor for the immune escape mechanism. Altogether, we propose nobiletin as a modulator of tumor microenvironment for cancer immunotherapy.

## 1. Introduction

Lung cancer is one of the most important cancer types, which constitutes an 18% death rate worldwide. Similarly, non-small cell lung cancer (NSCLC) is a subdivision of lung cancer that has recently recorded a survival rate of around 15% [1,2]. Patients who suffer from NSCLC struggle with specific chemotherapeutic drugs due to drug resistance and target specificity. This chemotherapy option also results in some serious side effects [3,4]. Therefore, although multiple approaches to treat NSCLC exist, such as radiation therapy, chemotherapy, targeted therapy, and surgery, the outcome of treatment was disappointing. These unsatisfactory results directed researchers to study more about the role of novel agents, such as natural compounds [5,6]. The added advantage of natural compounds is the ability to aim multiple targets with fewer side effects than non-natural candidate drugs [7,8].

Nobiletin (5,6,7,8,3′,4′-hexamethoxyflavone) is a natural flavonoid extracted from citrus peels with various functions, including anti-inflammatory [9] and anti-cancer activities [10,11]. Some studies on nobiletin showed that its mode of action is through regulating signal transduction and activator of transcription 3 (STAT3), which is a transcription factor functioning as an oncogene during tumorigenesis [12,13]. Nobiletin also acts against NSCLC cells by inducing apoptosis in A549 cells in vitro and in vivo [14], and it can inhibit epithelial-mesenchymal transition through TGF-β1/Smad3 signaling [15]. In H460 and H1299 NSCLC cells, nobiletin induced cell cycle arrest and apoptosis by regulating multiple proteins involved in tumor cell proliferation [16]. It also showed anti-inhibitory action against cigarette carcinogen NNK-induced lung tumorigenesis [17]. Similarly, nobiletin showed synergistic effects on NSCLC cells along with two other chemotherapeutic drugs, carboplatin and paclitaxel [18]. Hence, a deeper study on nobiletin against NSCLC will be an interesting topic in the development of natural compound-based therapeutics. 

Programmed cell death ligand-1 (PD-L1) encoded by cluster of differentiation 274 gene (*CD274*) is a transmembrane protein present on the outer membrane surface of antigen-presenting cells. It specifically binds to the inhibitory receptor, programmed cell death-1 (PD-1), which is present on the surfaces of lymphocytes, myeloid cells, T-cells, and B-cells, taking part in immune activities [19]. PD-L1/PD-1 interaction also plays a vital role in tumor immune escape through its immunosuppressive effect by suppressing the proliferation and cytotoxicity of T-cells as well as cytokine production and their release [20]. Many studies also show that PD-L1 is overexpressed in various cancer types, and it directs tumor development and progression. These observations, therefore, justify that the overexpression of PD-L1 facilitates cancer cells to undergo immune escape [21,22,23]. Furthermore, various oncogenic transcription factors, such as nuclear factor kappa-light-chain-enhancer of activated B-cells (NF-κB), hypoxia-inducible factor, or signal transduction and activator of transcriptions (STATs) regulate transcriptional regulation of PD-L1 expression, whereas tumor protein 53 (p53) plays a vital role where PD-L1 expression is inversely related to p53 expression [24,25]. A recent study showed that bioactive natural gallic acid inhibited PD-L1/PD-1 interaction by regulating epidermal growth factor receptor (EGFR)/p53 signaling in NSCLC cells [26].

EGFR is a transmembrane receptor whose overexpression is associated with approximately 40–89% of NSCLC cells [27]. Even though targeting EGFR with tyrosine kinase inhibitor is a good approach against NSCLC, few of them were ineffective regarding tumor response, including Gefitinib [28]. Elevated levels of EGFR enable signal transduction in the cell cytoplasm, thereby promoting tumorigenesis by contributing to the transformation of cellular phenotypes for cancer cell proliferation and survival [29]. Generally, blocking of EGFR signals toward its downstream targets by chemotherapeutic drugs determines the tumor fate. Additionally, the Janus kinase (JAK)–STAT pathway follows usual signaling from EGFR, where the ligand attaches to the transmembrane EGFR, thereby activating a cascade of phosphorylation responses. These reactions consist of the phosphorylation of JAKs, the receptor cytoplasmic tail, and the STAT transcription factors that phosphorylate STATs, thereby directing its localization to the nucleus to facilitate DNA binding and, therefore, gene regulation [30]. JAK2/STAT3 is considered a key signaling molecular pathway that promotes tumor cell growth and progression and participates in tumor metastasis by targeting molecular signals that regulate many of the cancer hallmarks [31]. Hence, targeting JAK2/STAT3 through EGFR signaling in NSCLC would be a good approach for natural compound-based therapeutics.

MicroRNAs (miRNAs) can inhibit mRNA translation and promote the degradation of mRNA, thereby resulting in the post-transcriptional alteration of gene expression [32]. These miRNAs also take part in the tumor immune system by their interaction with PD-L1. Therefore, among miRNAs, miR-34a is associated with PD-L1 expression in response to p53-dependent anti-tumor activities [26]. miR-197 is another miRNA, which is negatively associated with PD-L1 expression, which is indirectly regulated by the CDC28 protein-kinase regulatory subunit 1B (CKS1B) and STAT3 expression in NSCLC cells [33].

In this study, we investigated the evasion of immunosuppression activity of the natural flavonoid, nobiletin, in NSCLC cells—A549, H292, and H460. We also analyzed the molecular as well as miRNA signaling behind the role of nobiletin in evasion of immunosuppression. Furthermore, we examined its ability to suppress immune escape through the release of interferon (IFN)-γ in NSCLC cells.

## 2. Results

### 2.1. Nobiletin Inhibits PD-L1 Expression in NSCLC Cells

**Figure 1:**
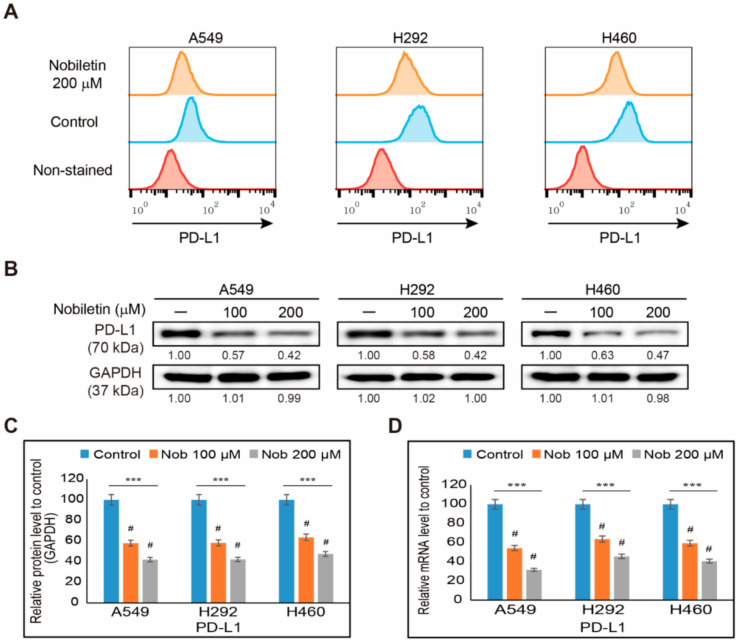
Nobiletin suppresses PD-L1 expression. (**A**) Flow cytometry analysis showing the surface PD-L1 expression from A549, H292, and H460 cells after treatment with 200 µM nobiletin for 48 h. (**B**) Western blotting of total proteins isolated from A549, H292, and H460 cells after 48 h treatment with 100 and 200 µM nobiletin. Results show the expression of PD-L1 proteins. (**C**) Representative expression of PD-L1 proteins was quantified by densitometry and normalized to GAPDH protein. Controls proteins were set to 100%. All data repeated three times for confirmation. *** *p* < 0.001 (ANOVA test). # *p* < 0.001 vs. control. (**D**) Real-time qPCR PD-L1 gene expression (*CD274*). The representative expression of PD-L1 mRNA is shown. Obtained Cp values were normalized to GAPDH mRNA and controls were set to 100%. *** *p* < 0.001. (ANOVA test), # *p* < 0.001 vs. control. Nob: Nobiletin.

To evaluate the ability of nobiletin to induce cell death, we analyzed the cell proliferation of A549, H292, and H460 NSCLC cells in the presence of increasing concentrations of nobiletin for 48 h (Figure S1). From these results, we considered 200 µM nobiletin at an IC_50_ dosage, and a concentration of 100 µM or 200 µM nobiletin was used for further studies. To determine the evasion of immunosuppression effect of nobiletin, we first analyzed the inhibition of PD-L1 expression by nobiletin in NSCLC cells—A549, H292, and H460. At first, we analyzed the surface expression of PD-L1 in all three NSCLC cells upon nobiletin treatment and found an inhibition in the surface expression of PD-L1 by nobiletin (Figure 1A). These results suggested the suppression of PD-L1 by nobiletin begin from surface level. So, we then checked the expression of protein levels of PD-L1 using Western blotting. Results showed a concentration-dependent inhibition of PD-L1 expression by nobiletin in these NSCLC cells (Figure 1B). Nobiletin at a concentration of 200 µM significantly downregulated PD-L1 levels by more than 50% in all three NSCLC cells (Figure 1C). This result suggested the possible role of nobiletin in evasion of immunosuppression through PD-L1 regulation. To confirm this result, we analyzed PD-L1 mRNA expression in NSCLC cells with or without nobiletin treatment (Figure 1D). Nobiletin also significantly suppressed the transcriptional analysis of the PD-L1 gene (*CD274*) expression, which suggested the role of nobiletin in PD-L1 inhibition, suggesting the possible evasion of the immunosuppression ability of nobiletin against A549, H292, and H460 NSCLC cells. 

### 2.2. Nobiletin Suppresses EGFR/JAK2/STAT3 Signaling Cascade in NSCLC Cells

**Figure 2:**
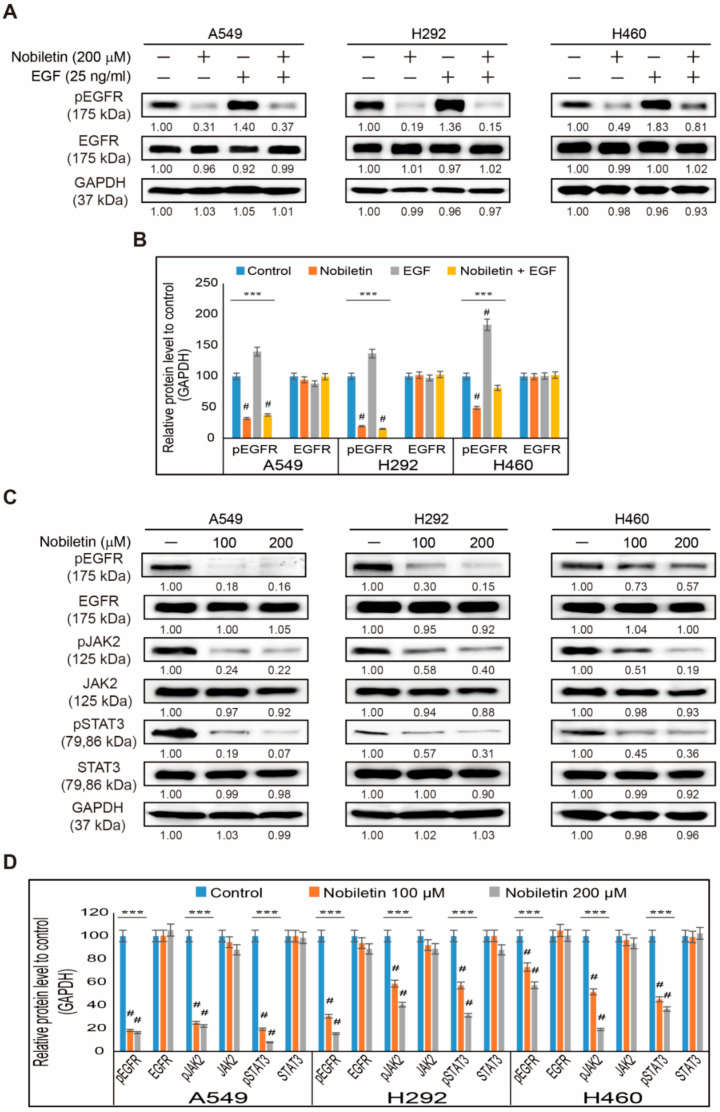
Nobiletin inhibits EGFR/JAK2/STAT3 signaling. (**A**) Western blotting showing the expression levels of phosphorylated EGFR and total EGFR proteins isolated from A549, H292, and H460 cells pretreated with 200 µM nobiletin for 48 h followed by the treatment with recombinant EGF (25 ng/mL) for 1 h. (**B**) Representative expression of pEGFR and EGFR proteins was quantified by densitometry and normalized to GAPDH protein. Controls proteins were set to 100%. All data repeated three times for confirmation. *** *p* < 0.001 (ANOVA test). # *p* < 0.001 vs. control. (**C**) Western blotting of total proteins isolated from A549, H292, and H460 NSCLC cells following 48 h treatment with 100 and 200 µM nobiletin. Results show expression levels for pEGFR, EGFR, pJAK2, JAK2, pSTAT3, and STAT3. (**D**) Representative expression of proteins was quantified by densitometry and normalized to GAPDH protein. Control proteins were set to 100%. All data were confirmed after repeating the experiment three times. *** *p* < 0.001 (ANOVA test).# *p* < 0.001 vs. control.

We found the inhibition of PD-L1 expression upon nobiletin treatment in the three NSCLC cells: A549, H292, and H460. We also analyzed the molecular pathway responsible for the inhibition ability of nobiletin. First, we analyzed the binding of nobiletin to the EGFR, as it is well-known in other cancer cells. Then, we used a treatment with recombinant human epidermal growth factor (EGF) following pretreatment with nobiletin and found an elevation in the expression of phosphorylated EGFR with recombinant treated cells, which were significantly decreased by nobiletin treatment (Figure 2A). Total EGFR levels remained the same upon nobiletin treatment in all three NSCLC cells (Figure 2B). The results, therefore, suggested the binding of nobiletin to the EGFR, resulting in the suppression of EGFR activation. Therefore, we analyzed the JAK2/STAT3 pathway, a key downstream target of EGFR (Figure 2C). The obtained results indicated that nobiletin significantly downregulated the phosphorylated expressions of EGFR, JAK2, and STAT3 without altering their total protein expressions to a large extent (Figure 2D). These results thus suggested that PD-L1 regulation of nobiletin occurs through EGFR/JAK2/STAT3 signaling. To confirm the role of phosphorylated STAT3 on PD-L1 expression, we estimated the expression levels of EGFR/STAT3/PD-L1 protein signaling upon treatment of an EGFR-tyrosine kinase inhibitor, AG1478 with or without nobiletin (Figure S2A). These results confirmed that STAT3 plays a vital role in the expression of PD-L1, and nobiletin inhibits PD-L1 expression by suppressing STAT3 activation (Figure S2B). 

### 2.3. Nobiletin Downregulates PD-L1 Expression through STAT3 Signaling

**Figure 3:**
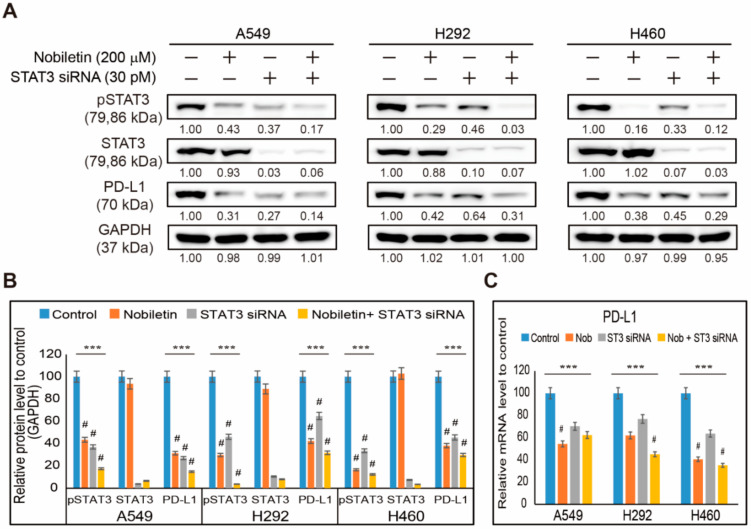
Nobiletin inhibits PD-L1 expression levels through STAT3. (**A**) Western blotting of A549, H292, and H460 cells was treated with STAT3 siRNA (30 pM) for 24 h, then treated with 200 µM nobiletin for 48 h. Diagrams show the expression patterns of phosphorylated STAT3 and PD-L1 proteins. (**B**) Representative expression of proteins was quantified by densitometry and normalized to GAPDH protein. Controls proteins were set to 100%. All data repeated three times for confirmation. *** *p* < 0.001 (ANOVA test). # *p* < 0.001 vs. control. (**C**) Real-time qPCR of PD-L1 gene expression (*CD274*) upon treatment with 30 pM STAT3 siRNA for 24 h followed by 200 µM nobiletin for 48 h in A549, H292, and H460 cells. The representative expression of PD-L1 mRNA is shown; Cp values were then normalized to GAPDH mRNA. Controls are set to 100. *** *p* < 0.001. (ANOVA test), # *p* < 0.001 vs. control. ST3: STAT3; Nob: Nobiletin.

Previously, we found that nobiletin can inhibit the expression levels of PD-L1 and phospho-STAT3. So, we analyzed the relation between STAT3 and PD-L1 in the presence of nobiletin in NSCLC cells. First, we silenced the expression of STAT3 using STAT3 siRNA and then treated the cells with nobiletin to analyze PD-L1 expression (Figure 3A). The results showed an inhibition of phospho-STAT3 and PD-L1 expression upon STAT3 siRNA treatment, which was, further, significantly downregulated by nobiletin treatment (Figure 3B). These results suggested the role of STAT3 in the inhibition of PD-L1 by nobiletin. Therefore, to confirm the results, we analyzed PD-L1 mRNA expression after treatment with STAT3 siRNA and nobiletin (Figure 3C). These results showed a suppression of PD-L1 mRNA upon STAT3 siRNA treatment, which was further inhibited by nobiletin treatment. These results indicate the role of STAT3 in the inhibition of PD-L1 expression in NSCLC cells by nobiletin treatment. 

### 2.4. Nobiletin Inhibits p53-MDM2 Signaling in NSCLC Cells

**Figure 4:**
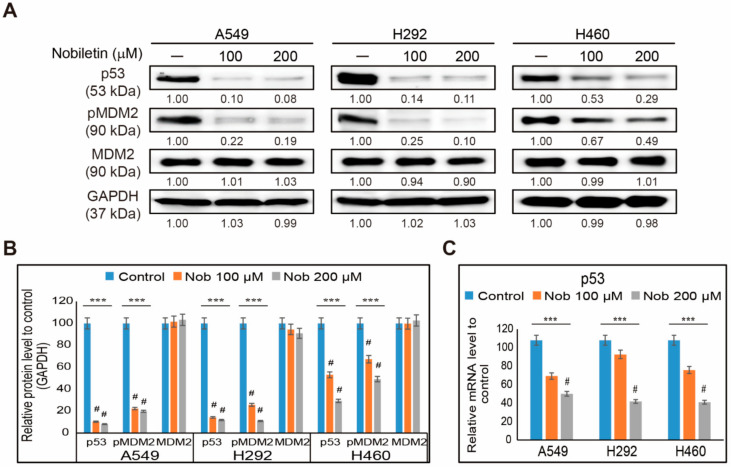
Nobiletin suppresses p53-MDM2 expressions. (**A**) Western blotting of total proteins isolated from A549, H292, and H460 cells after 48 h treatment with 100 and 200 µM nobiletin showed the expression of p53, MDM2, and phospho-MDM2 proteins. (**B**) Representative expression of proteins was quantified by densitometry and normalized to GAPDH protein. Control proteins were set to 100%. All data repeated three times for confirmation. *** *p* < 0.001 (ANOVA test). # *p* < 0.001 vs. control. (**C**) Real-time qPCR of the expression of p53 genes (*TP53*) in NSCLC cells after treatment with 100 and 200 µM nobiletin for 48 h. The representative expression of p53 mRNA is shown; Cp values were then normalized to GAPDH mRNA. Controls are set to 100. *** *p* < 0.001. (ANOVA test), # *p* < 0.001 vs. control. Nob: Nobiletin.

We observed suppression in EGFR/JAK2/STAT3 signaling and PD-L1 signaling in NSCLC cells by nobiletin treatment. So, we assumed that p53 contributed to the regulation between STAT3 and PD-L1. Therefore, we determined the expression levels of p53 and its negative regulator, MDM2, upon nobiletin treatment in A549, H292, and H460 cells (Figure 4A). The obtained results showed an opposite pattern from our assumption of p53 expression. Increasing concentrations of nobiletin downregulated the expression levels of p53 along with the phosphorylated expressions of MDM2 (Figure 4B). This result thus suggested a possibility of the p53-independent inhibition of PD-L1 by nobiletin. In some cases, there were little effects of p53 expression in protein and mRNA levels, which proposed a key effect in the regulation of p53 expression. Therefore, we analyzed the p53 mRNA expression in NSCLC cells after nobiletin treatment (Figure 4C). The results also showed significant inhibition in p53 mRNA expression by nobiletin treatment, which suggested that nobiletin induces PD-L1 inhibition in a p53-independent manner. 

### 2.5. Nobiletin Inhibits PD-L1 Expression in a p53-Independent Manner

**Figure 5:**
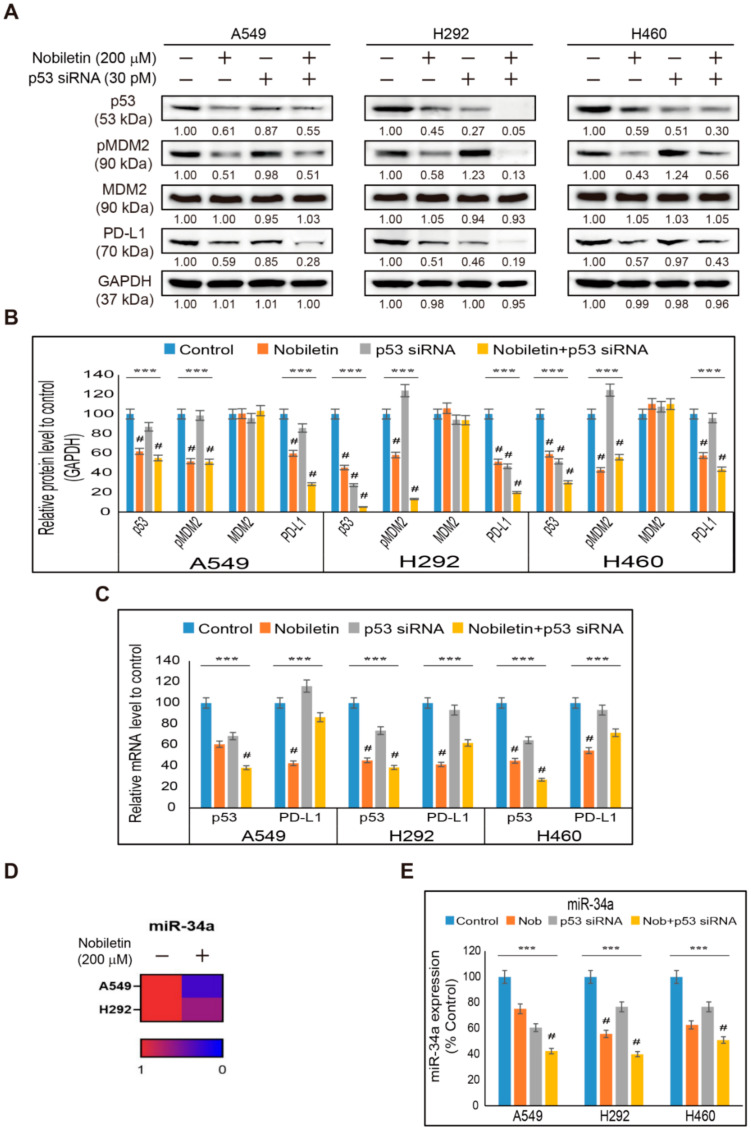
p53-Independent inhibition of PD-L1 by nobiletin. (**A**) Western blotting analysis of A549, H292, and H460 cells pretreated with p53 siRNA (30 pM) for 24 h, then treated with 200 µM nobiletin for 48 h shows the expression patterns of p53, MDM2, pMDM2, and PD-L1 proteins. (**B**) Representative expression of proteins was quantified by densitometry and normalized to GAPDH protein. Control proteins were set to 100%. All data repeated three times for confirmation. *** *p* < 0.001 (ANOVA test). # *p* < 0.001 vs. control. (**C**) Real-time qPCR of p53 (*TP53*) and PD-L1 gene (*CD274*) expressions upon treatment with 30 pM p53 siRNA for 24 h followed by 200 µM nobiletin for 48 h in A549, H292, and H460 cells. The representative expression of p53 and PD-L1 mRNA is shown; Cp values were then normalized to GAPDH mRNA. Controls are set to 100. *** *p* < 0.001. (ANOVA test), # *p* < 0.001 vs. control. (**D**) Heat map displaying the fold changes related to the mean expression of miR-34a in control and 200 µM nobiletin-treated A549 and H292 cells. (**E**) Real-time qPCR analysis of miR-34a expression upon treatment with 30 pM p53 siRNA for 24 h followed by 200 µM nobiletin for 48 h in A549, H292, and H460 cells. Representative expression of miR-34a mRNA is shown. Cp values were then normalized to U6 mRNA. Controls are set to 100. *** *p* < 0.001. (ANOVA test), # *p* < 0.001 vs. control. Nob: Nobiletin.

We observed inhibition in the expression of p53 in NSCLC cells by nobiletin treatment. To prove the observed p53-independent inhibition of PD-L1 expression, we treated NSCLC cells with p53 siRNA or p53 inhibitor (Pifithrin-α; PFT-α) along with nobiletin. The addition of p53 siRNA showed an inhibition of the expression of p53 and phospho-MDM2 with an altered expression of PD-L1 (Figure 5A). Nobiletin treatment along with p53 siRNA furthermore inhibited the expression of p53, pMDM2, and PD-L1, along with unchanged total MDM2 expressions in NSCLC cells (Figure 5B). To confirm the activity of p53 siRNA along with nobiletin, we analyzed *TP53* and *CD274* gene expression in mRNA levels. We also obtained results that showed a similar pattern as seen in the protein level (Figure 5C). Additionally, to double-check the absence of p53 in PD-L1 inhibition, we treated NSCLC cells with PFT-α, a specific inhibitor of p53 transcription activity and its combination treatment with nobiletin, and then analyzed the protein levels of p53, phospho-MDM2, and PD-L1 expression (Figure S3A). The gained results also showed a similar pattern with p53 siRNA activity, where the combination of nobiletin and PFT-α further inhibited p53 expression as we observed in PFT-α alone (Figure S3B). These results proved the p53-independent inhibition of PD-L1 by nobiletin. For further confirmation of p53-independent action, we demonstrated a miRNA analysis in A549 and H292 cells after treatment with 200 µM nobiletin. The analysis produced the expression of several miRNAs. Among them, miR-34a was considered a key miRNA that co-relates p53 inhibition with PD-L1 expression. Our results also showed an inhibition of the levels of miR-34a in A549 and H292 cells upon nobiletin treatment (Figure 5D). Therefore, to confirm the miR-34a expression after nobiletin treatment and its p53-independent mechanism, we analyzed miR-34a levels after treatment with p53 siRNA and nobiletin in A549, H292, and H460 cells. The obtained results showed an inhibition in the expression of miR-34a in p53 siRNA-treated cells, which was further inhibited by nobiletin treatment (Figure 5E). These results proved the p53-independent suppression of PD-L1 expression by nobiletin.

### 2.6. miR-197 Involved in the Suppression of STAT3-PD-L1 Signaling by Nobiletin in NSCLC Cells

**Figure 6:**
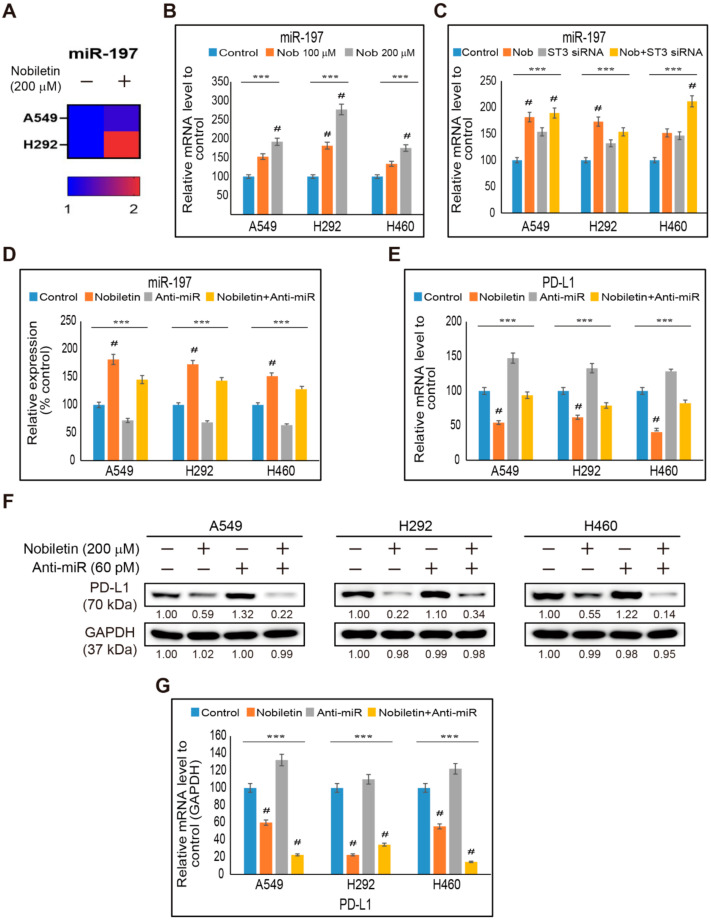
Nobiletin upregulates miR-197 and regulates PD-L1 expression. (**A**) Heat map displaying the fold changes related to the mean expression of miR-197 in non-treated and nobiletin-treated A549 and H292 cells. (**B**) Real-time qPCR of miR-197 expression upon treatment with 100 and 200 µM nobiletin for 48 h in A549, H292, and H460 cells. The representative expression of miR-197 mRNA is shown; Cp values were then normalized to U6 mRNA. Controls are set to 100. *** *p* < 0.001. (ANOVA test), # *p* < 0.001 vs. control. (**C**) Real-time qPCR of miR-197 expression upon treatment with 30 pM STAT3 siRNA for 24 h followed by 200 µM nobiletin for 48 h in A549, H292, and H460 cells. The representative expression of miR-197 mRNA is shown; Cp values were then normalized to U6 mRNA. Controls are set to 100. *** *p* < 0.001. (ANOVA test), # *p* < 0.001 vs. control. (**D**) Real-time qPCR of miR-197 expression upon treatment with 60 pM anti-miR for 24 h, followed by 200 µM nobiletin for 48 h in A549, H292, and H460 cells. The representative expression of miR-197 mRNA is shown; Cp values were then normalized to U6 mRNA. Controls are set to 100. *** *p* < 0.001. (ANOVA test), *p* < 0.001 vs. control. (**E**) Real-time qPCR of PD-L1 expression upon treatment with 60 pM anti-miR for 24 h followed by 200 µM nobiletin for 48 h in A549, H292, and H460 cells. The representative expression of miR-197 mRNA is shown; Cp values were then normalized to GAPDH mRNA. Controls are set to 100. *** *p* < 0.001. (ANOVA test), # *p* < 0.001 vs. control. (**F**) Western blotting of A549, H292, and H460 cells pretreated with anti-miR (60 pM) for 24 h and then treated with 200 µM nobiletin for 48 h shows the expression patterns of PD-L1 proteins. (**G**) Representative expression of PD-L1 proteins was quantified by densitometry and normalized to GAPDH protein. Control proteins were set to 100%. All data repeated three times for confirmation. *** *p* < 0.001 (ANOVA test). # *p* < 0.001 vs. control. ST3: STAT3; Nob: Nobiletin.

We found that p53 was not taking part in the inhibition of PD-L1 expression by nobiletin treatment. We also found the inhibition of phospho-STAT3 and PD-L1 expression in NSCLC cells by nobiletin treatment, suggesting the possible role of miR-197 in the inhibition of STAT3 and PD-L1 by nobiletin. miRNA analysis showed that the addition of nobiletin increased the fold change of miR-197 to non-treated control cells (Figure 6A). Hence, we confirmed the elevation in the expression of miR-197 with the increasing concentration of nobiletin in NSCLC cells (Figure 6B). The results indicated the role of miR-197 in the PD-L1-dependent evasion of immunosuppression effect of nobiletin. To check the role of STAT3 in the expression of miR-197 by nobiletin treatment, we analyzed the expression of miR-197 with or without STAT3 siRNA and nobiletin (Figure 6C). The obtained results also showed an elevation in the expression of miR-197 with STAT3 siRNA treatment, which was further upregulated by nobiletin treatment. Hence, STAT3/miR-197 signaling was proposed to play an important role in PD-L1 inhibition by nobiletin. To confirm this fact, we used a microRNA inhibitor (anti-miR) along with nobiletin treatment to analyze the expression of miR-197 (Figure 6D). The results obtained showed the inhibition of miR-197 expression in anti-miR-treated cells, whereas further addition of nobiletin upregulated its expression. These results also suggested the role of miR-197 in the regulation of PD-L1 expression upon nobiletin treatment. Therefore, we confirmed this observation as well by checking the expression of PD-L1 mRNA in NSCLC after treatment with anti-miR and nobiletin. The results showed an increase in the expression of PD-L1 mRNA upon anti-miR treatment, which was inhibited by nobiletin treatment (Figure 6E). Thus, to confirm the relationship between miR-197 and PD-L1 expression, we further analyzed PD-L1 protein expression in the presence of anti-miR and nobiletin (Figure 6F). The results also showed similar results, as seen with PD-L1 mRNA, where the addition of anti-Mir upregulated the expression of PD-L1, which was significantly downregulated by nobiletin treatment (Figure 6G). These results indicated that nobiletin possessed the evasion of immunosuppression abilities by regulating miR-197/PD-L1 signaling through STAT3 expression. 

### 2.7. Combination Effect of PD-L1 Expression by Nobiletin with PD-1 Blockade in NSCLC Cells

**Figure 7:**
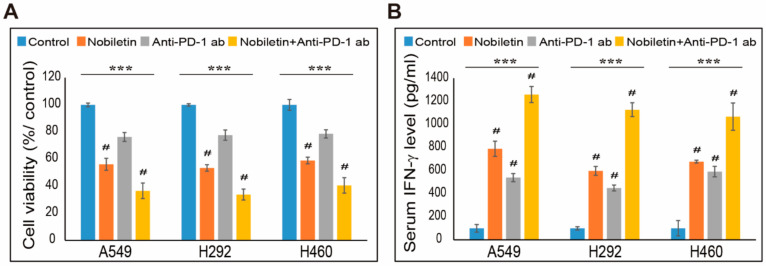
Nobiletin exhibits a combination effect with anti-PD-1 mAb in an NSCLC–PBMC co-culture system. (**A**) Cell survival analysis of NSCLC cells A549, H292, and H460 co-cultured with PBMCs were examined after treatment with 200 µM nobiletin, anti-PD-1 mAb (5 µg/mL), or both nobiletin and anti-PD-1 mAb for 48 h. *** *p* < 0.001 (ANOVA); # *p* < 0.001 vs. control. (**B**) Analysis of IFN-γ release from the co-culture system were determined by ELISA after treatment with 200 µM nobiletin, 5 µg/mL anti-PD-1 mAb, or their combination for 48 h. *** *p* < 0.001 (ANOVA); # *p* < 0.001 vs. control.

**Figure 8:**
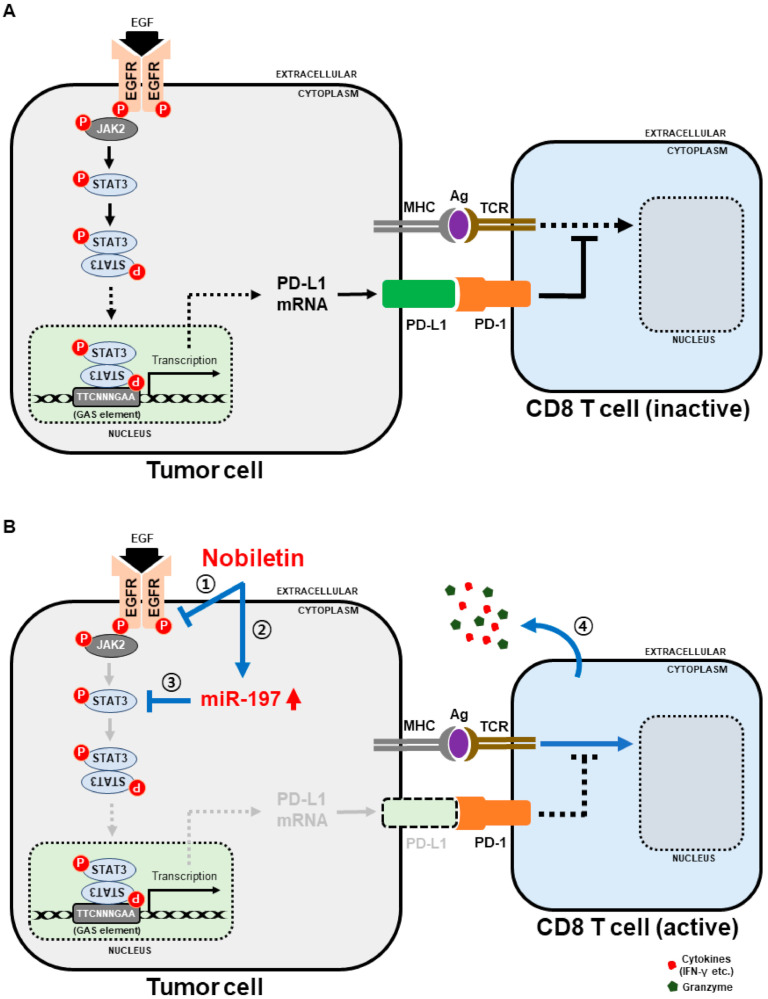
The molecular regulatory mechanism behind role of nobiletin in evasion of immunosuppression in NSCLC cells. (**A**) Tumor tolerance when PD-1/PD-L1 interaction. (**B**) Tumor clearance when the interaction is blocked.; **①**, **②**, and **③** Nobiletin inhibits the EGFR-mediated JAK2/STAT3 signaling pathway and thereby, PD-L1 inhibition through miR-197 regulation. **④** CD8 T cells sorted from PBMC induce NSCLC cell-apoptosis by secreting cytokines (IFN-γ, etc.) and granzymes.

We determined that nobiletin can inhibit PD-L1 expression in NSCLC cells. However, its capability to suppress immune escape not only depends on PD-L1 but the ones present in immune cells. Therefore, to analyze these processes, we used a co-culture system with NSCLC cells and peripheral blood mononuclear cells (PBMC) treated with or without nobiletin or nivolumab, an anti-PD-1 monoclonal antibody (mAb) or their combination. First, we evaluated the cell toxicity induced by these drugs and their combination in co-culture systems. The obtained results showed a cytotoxic effect in all three NSCLC cells in nobiletin and PD-1 mAb-treated cells (Figure 7A). The combination of these drugs also induced more cell death than their individual concentrations. These results thus suggested that nobiletin reduced survival signals of PD-L1 and activated T-cell-mediated immune responses. To confirm this hypothesis, we evaluated PBMC cytokine secretion by performing IFN-γ enzyme-linked immunosorbent assay (ELISA). The results indicated that the release of cytokines was elevated in PD-1 mAb than in control cells, whereas the combination with nobiletin further increased cytokine secretion in the supernatant compared with nobiletin alone (Figure 7B). These results suggested the role of nobiletin in the evasion of immunosuppression by targeting the PD-1/PD-L1 blockade. In order to confirm the IFN-γ released from T-cell in cytotoxicity to NSCLC cells, we analyzed IFN-γ release in NSCLC upon the combination treatment with PD-1 mAb and nobiletin (Figure S4). The obtained results showed a non-significant result in NSCLC cells which clearly suggested that release of IFN-γ occurred from PBMC-NSCLC co-culture system as a result of PD-1/PD-L1 blockade. Altogether, nobiletin inhibited PD-L1 expression by regulating EGFR/Jak2/STAT3 and miR-197 signaling. It also exhibits the ability of nobiletin in the evasion of immunosuppression by targeting the PD-1/PD-L1 blockade (Figure 8).

## 3. Discussion

Cancer treatment using drugs mainly depends on how the drug acts against cancer cells without affecting the behavior of normal cells. This observation is considered the most common drawback of chemotherapeutic drugs, as they destroy normal cells along with cancer cells. Due to the side effects and multidrug resistance of chemotherapeutic drugs against NSCLC cells, many drugs, and their combination treatment, patients are still suffering because of their limited abilities [34,35]. Therefore, studies on natural compounds are an engaging topic, as it is considered one of the target therapies for various cancers with fewer side effects [36,37]. Nobiletin is a natural flavonoid found to have anticancer activities against various cancers [38,39,40]. Even though nobiletin has several anticancer activities, its role in the evasion of immunosuppression has not yet been studied. 

EGFR is a famous receptor found to be overexpressed in most NSCLC cells, and its regulation is essential to manage tumor progression during targeted therapy [41]. The results from experiments on recombinant EGF also showed that nobiletin binds to EGFR and suppresses EGFR phosphorylation, thereby blocking its downstream signals. Nobiletin also results in the suppression of JAK2 phosphorylation, which is involved in different cell signaling reactions to promote tumorigenesis [42]. Similarly, nobiletin successfully suppressed the molecular signaling of EGFR/JAK2 and its interaction with other proteins, such as STAT3, thereby enhancing PD-L1 regulation. This function has been proven to have a role in tumor metastasis and immune escape [43]. STAT3 can bind to the PD-L1 promoter and proceed with the transcription process [44]. Nobiletin is well known for its binding towards EGFR and inhibits the phosphorylation of EGFR and thereby suppress the expression levels of the downstream targets associated with EGFR [13,45]. Hence, according to molecular signaling, nobiletin inhibits tumor progression by inhibiting the EGFR/JAK2/STAT3 pathway and PD-L1 expression in NSCLC cells. Suppression of PD-L1 in cancer cells by nobiletin gave a hint for its role in evasion of immunosuppression activity. Therefore, we conducted further studies to determine that ability. 

PD-L1 regulation depends on p53 activation, where p53 negatively co-relates with PD-L1 expression [25]. The activation of a miRNA, miR-34a, regulates this mechanism [46]. Studies on the role of p53 in immune evasion are still ongoing, as some studies showed that p53 has a surprising role in the intrinsic PD-1 regulation of cancer cells [47]. Additionally, although p53 is considered an important factor in PD-L1 regulation, our results exhibit the opposite, as the addition of nobiletin inhibited p53 expression as well as its negative regulator, MDM2. These results, therefore, propose that a p53-independent mechanism is involved in the activity of nobiletin in NSCLC cells. Hence, we confirmed this activity by inhibiting p53 expression using p53 siRNA and specific p53 inhibitors. Then, we treated NSCLC cells with nobiletin to analyze whether nobiletin upregulated the expression of p53. The results also indicated a p53-independent regulation of PD-L1 expression by nobiletin. As the expression of p53 on nobiletin treatment blocked our hypothesis, we analyzed miRNA sequencing to direct further studies. Among the miRNAs, miR-34a was associated with PD-L1 expression through its binding with 3′UTR of the *PD-L1* mRNA that directs PD-L1 inhibition [46]. Therefore, to support the p53 inhibition, we obtained a reduction in the fold change of miR-34a upon nobiletin treatment, which strongly confirmed the hypothesis of p53-independent PD-L1 inhibition. 

miRNAs play vital roles in cancer hallmarks by regulating various signaling pathways [32]. Many miRNAs regulate PD-L1 expression, including miR-138, miR-197, miR-200, and miR-513. Among them, miR-197 plays a key role in tumor survival of different cancer types, including tongue cancer [48], thyroid cancer [49], lung cancer [50], and pancreatic cancer [51]. It has also been negatively associated with PD-L1 expression, where the elevation of miR-197 inhibits PD-L1 expression by regulating CKS1B/STAT3 signaling in NSCLC cells [33]. Our previous results showed an inhibition in the expression of PD-L1 and phospho-STAT3 by nobiletin treatment in NSCLC cells. Additionally, we confirmed the relationship between STAT3 and PD-L1 by silencing STAT3 using STAT3 siRNA and found a suppressed expression of PD-L1, which was further downregulated by the addition of nobiletin. These results propose a possible role of miR-197 in the activity of nobiletin in NSCLC cells. Therefore, to check the activity of miR-197, we analyzed all miRNAs by sequencing and found a slight increase in the fold change of miR-197 expression by nobiletin in A549 cells, whereas an approximately two-fold change in miR-197 was observed in H292 cells upon nobiletin treatment. These results propose a possible mechanism of nobiletin by suppressing the expression of STAT3 and PD-L1, thus enhancing miR-197 signaling. We then confirmed the elevation of miR-197 signaling by nobiletin using miR-197 expression analysis, as the role of STAT3 in this mechanism has been confirmed using STAT3 siRNA. The silencing of STAT3 increased the expression of miR-197. However, further addition of nobiletin significantly increased the expression of miR-197. The negative regulation of miR-197 on PD-L1 expression was also confirmed using a miRNA inhibitor, anti-Mir, where the inhibition of miR-197 enhanced PD-L1 expression. From the results, nobiletin successfully decreased the elevated PD-L1 expression. Hence, it is evident that nobiletin takes part in immunosuppression by inhibiting PD-L1 expression and by regulating STAT3 and miR-197 signaling. 

The natural flavonoid nobiletin shows promising inhibitory activity in the expression of PD-L1 in NSCLC cells. Therefore, the key role of PD-L1 in immune resistance depends on the blockade of its binding toward PD-1, which also plays a crucial role in immune inhibition due to its inhibitory effects on the function of lymphocytes when it binds to PD-L1 [52], so that tumor immune resistance from the cytotoxic cluster of differentiation CD8 T-cells can occur. This resistance is possible through the blockade of PD-1/PD-L1 interaction and inhibition of PD-L1, thereby leading CD8 T-cell immune responses to act against tumor cells [53]. Hence, PD-1/PD-L1-dependent immunotherapy is an effective method, and we found that nobiletin was successfully involved in this approach. We also observed the suppression of PD-L1 in NSCLC cells by nobiletin and evaluated its effect using the co-culture method with PBMC. We found that nobiletin exhibited combination effects with human monoclonal anti-PD-1 mAb, nivolumab, against cell proliferation inhibition in the NSCLC–PBMC co-culture system. The inhibition of both PD-1 and PD-L1 also showed a significant reduction in cancer cell viability, which shows the blockade on PD-1/PD-L1 interaction. Similarly, IFN-γ is a cytokine considered as a main stimulator of PD-L1 expression and plays a vital role in the tumor microenvironment through its secretion by inflammatory cells [54]. Nobiletin, along with anti-PD-1 mAb, significantly increased the level of IFN-γ in co-culture systems, which gave a strong proof for the immune response induced by nobiletin through the PD-1/PD-L1 blockade. Hence, nobiletin is proposed to be a good candidate drug for natural compound-based therapeutics in cancer immunotherapy against NSCLC cells and in order to confirm this, in vivo experimentation needed for further analysis that could confirm that nobiletin inhibits PD-L1 expression through mir-197 regulation instead of miR-34a. 

## 4. Materials and Methods

### 4.1. Antibodies and Cell Culture Reagents

RPMI-1640 medium, 0.05% trypsin–EDTA, and penicillin–streptomycin solution was obtained from Chemical Industry Co., Ltd. (Portland, OR, USA). Fetal bovine serum (FBS) and PFT-α (P4359) were also obtained from Sigma-Aldrich (Merck KGaA, St. Louis, MO, USA). Antibodies used for Western blotting, specifically phosphorylated EGFR (pEGFR; #3777), EGFR (#4267), phosphorylated JAK2 (pJAK2; (#3776), JAK2 (#3230), phosphorylated STAT3 (pSTAT3; #9145), STAT3 (#9139), p53 (#9282), phosphorylated MDM2 (#3521), MDM2 (#86934), and GAPDH (#2118) were bought from Cell Signaling Technology (Beverly, MA, USA). The antibody specific for PD-L1 (R30949) was obtained from NSJ Bioreagents (San Diego, CA, USA).

### 4.2. Cell Culture and Treatment

A549 (10185), H292 (21848), and H460 (30177) cell lines were purchased from the Korean Cell Line Bank (Jongno-gu, Seoul, Korea). These cell lines were then cultured and maintained in RPMI-1640 plus 10% FBS and 1% penicillin at 37 °C in 5% CO_2_. Subsequently, the medium was changed three times a week after cells were grown for up to 80% confluence and treated with nobiletin. Then, the setup was incubated at 37 °C for 48 h.

### 4.3. Cell Viability Assay

Cell viability was measured using a 3-(4,5-dimethylthiazol-2-yl)-2,5-diphenyltetrazolium bromide (MTT) assay. Cells were maintained in RPMI-1640 in 96-well culture plates at 3 × 10^3^ per well (density) for 24 h. Cells were again incubated with a new medium containing dimethyl sulfoxide (DMSO) as the vehicle control and then treated with various concentrations of nobiletin (50–300 μM) for 48 h. Subsequently, MTT (5 mg/mL) was added and incubated again for 4 h at 37 °C. The resulting formazan product was then dissolved in DMSO, after which an Ultra Multifunctional Microplate Reader (Tecan, Durham, NC, USA) was used to measure the absorbance at a wavelength of 560 nm. All measurements and experiments were conducted three times.

### 4.4. Flow Cytometry Analysis of Surface PD-L1 Expression

After cultured cells were washed with pre-chilled PBS, cell pellets were incubated with 10% BSA on ice for 20 min. The following antibodies (1/200 diluted) were used for staining on ice for 30 min: FITC anti-human CD274 (B7-H1, PD-L1) (374509; Biolegend, San Diego, CA, USA). Stained cells were washed with pre-chilled PBS. The stained cells were washed with 1 mL of prewarmed staining buffer. Cytometry analysis was performed using a FACS Calibur flow cytometer (BD Biosciences, San Jose, CA, USA).

### 4.5. Western Blotting Analysis

Cells are allowed to grow up to 70~89% confluence and protein samples were isolated from untreated (control) or nobiletin-treated A549, H292, and H460 cells after aspirating dead cells and then allowed for lysis using a radioimmunoprecipitation (RIPA) lysis buffer (20–188; EMD Millipore), containing protease and phosphatase inhibitors. The concentration of proteins was measured using Bradford’s method (Thermo Fisher Scientific, Inc., Waltham, MA, USA). Next, the same amounts of isolated protein samples (100 μg/well) were resolved with sodium dodecyl sulfate-polyacrylamide gel (10–15%) electrophoresis. The separated proteins were then transferred onto nitrocellulose membranes, followed by blocking with 5% skim milk (BD Biosciences, San Jose, CA, USA) in TBS-T buffer (20 mM Tris-HCl (Sigma-Aldrich; Merck KGa A), pH 7.6, 137 mM NaCl (Formedium, Norfolk, UK; NAC03), and 0.1 × Tween20 (Scientific Sales, Inc. OakRidge, TN, USA)) for 1 h. The membranes were then incubated overnight at 4 °C in a shaker with specific primary antibodies diluted in 5% bovine serum albumin (EMDMillipore). Then, membranes were washed with TBS-T and incubated with HRP-conjugated secondary antibodies for 1 h at room temperature. Finally, the detection was performed using a Femto Clean Enhanced Chemiluminescence Solution Kit (77449; GenDEPOT, Katy, TX, USA) in a LAS-4000 imaging device (Fujifilm, Tokyo, Japan). Quantifications were carried out using ImageJ software (version 1.8.0_172; National Institutes of Health, Bethesda, MD, USA).

### 4.6. Quantitative Real-Time Polymerase Chain Reaction (qPCR)

Total RNA was isolated using RNeasy Mini Kit (Qiagen GmbH, Hilden, Germany) and then quantified using a spectrophotometer at 260 nm. Subsequently, a thermal cycler (C1000 Thermal Cycler; Bio-Rad, Hercules, CA, USA) was used to make cDNA from the total RNA using a first-strand cDNA synthesis kit (Bioneer, Daejeon, Korea) and oligo (dT) primers. PD-L1, p53, and GAPDH cDNA were amplified using an RT-PCR Premix Kit (Bioneer) with primers synthesized by Bioneer. Furthermore, the Light Cycler 480II (Roche) was used for qPCR as follows: 2 μL diluted cDNA was mixed with 10 μL TB Green Advantage Premix (Takara Bio, Japan) and 1-μL each of forward and reverse primers. The cycling conditions were as follows: 95 °C for 5 min for the initial denaturation, followed by 40 cycles of 95 °C for 40 s, 58 °C for 40 s, 72 °C for 40 s, and finally, extended for 5 min at 72 °C. All reactions were conducted three times and normalized to GAPDH, and quantifications were conducted using obtained Cp values.

### 4.7. Transfections of siRNA and miRNA

A549, H292, and H460 cells (1 × 10^5^ cells) were seeded in six-well plates and grown up to 50~60% confluence. The cells were then transfected with p53 siRNA (sc-29435), STAT3 siRNA (sc-29493) from Santa Cruz Biotechnology (Dallas, TX, USA), or anti-miR (microRNA inhibitor; AM 17000; Thermo Fisher Scientific, Inc., Waltham, MA, USA) using a Lipofectamine transfection reagent (Thermo Fisher Scientific, Inc., Waltham, MA, USA) for 24 h. Then, the cells were additionally treated with/without nobiletin for 48 h, and then proteins were isolated for analysis using Western blotting.

### 4.8. Small-RNA Sequence Analysis

First, total RNA was isolated from A549 and H292 cells treated with or without nobiletin using TRIzol reagent (Invitrogen, Carlsbad, CA, USA) according to the manufacturer’s instructions. Agilent 2100 Bioanalyzer used for assessing the RNA quality using the RNA 6000 Pico Chip (Agilent Technologies, Amstelveen, The Netherlands) and a NanoDrop-2000 Spectrophotometer system (Thermo Fisher Scientific, Waltham, MA, USA) used for RNA quantification. The construction of libraries for control and nobiletin-treated RNAs was performed using the NEBNext Multiplex Small-RNA Library Prep kit (New England BioLabs, Inc., Ipswich, MA, USA). For library construction, 1 µg total RNA from each sample was used to ligate the adaptors, and cDNA was synthesized using reverse transcriptase with specific primers. Subsequently, PCR was conducted for library amplification, and QIAquick PCR Purification Kit (Qiagen, Inc, Qiagen Str. Hilden, Germany) and AMPure XP beads (Beckmancoulter, Inc., Brea, CA, USA) were used for library purification. Agilent 2100 Bioanalyzer instrument for High-sensitivity DNA Assay (Agilent Technologies, Inc., 5301 Stevens Creek Blvd, CA, USA) used to analyze the yield and size distribution of small-RNA libraries. Then, a NextSeq-500 system was used as a way of single-end 75 sequencings (Illumina, San Diego, CA., USA) to produce high-throughput sequences. Then, a bowtie2 software tool was used to obtain a bam file for data analysis sequence reads. For the reference for mapping, a mature miRNA sequence was used, and the extraction of reading counts mapped on mature miRNA sequence from the alignment file was conducted by bedtools (v.2.25.0; Boston, MA, USA) and a statistical programming language, Bioconductor, which uses R (version 3.2.2; R Development Core Team, 2011; Seattle, WA, USA). Finally, read counts were used to estimating the miRNAs expression levels. The quantile normalization method was used to compare between samples, and miRWalk 2.0 was conducted for the miRNA target studies.

### 4.9. NSCLC Cells and PBMCs Co-Culture Experiments

A549, H292, and H460 cells (5 × 10^4^ cells) were seeded in 24-well plates until 70–80% confluence under a cell culture condition. To isolate human PBMCs from peripheral blood donated by healthy volunteers, FicollPaque density centrifugation as well as Lymphoprep™ and SepMate™−50 (Stemcell Technologies, Vancouver, Canada) were used. The acquired PBMCs were seeded to a co-culture system at a PBMCs/attached NSCLC cell ratio of 5:1. The wells were treated with 5 µg/mL of anti-PD-1 mAb (nivolumab, #A1307; BioVision, Milpitas, CA, USA) or nobiletin (200 µM) or their combinations for 48 h. Then, the spend media were collected to analyze the human IFN-γ level, while the NSCLC cells viability was determined by MTT assay. The human IFN-γ were evaluated using an ELISA kit (#430104; BioLegend, San Diego, CA, USA) according to the manufacturer’s protocol.

### 4.10. Statistical Analyses

All experiments were conducted three times, and the results were expressed as the mean ± standard error of the mean. Statistical analyses were conducted either using Student’s *t*-test or one-way analysis of variance (ANOVA). Tukey’s post hoc test was used to conduct one-way ANOVA. Subsequently, analyses were performed using SAS version 9.3 software (SAS Institute, Inc., Cary, NC, USA). *p* < 0.05; (*) indicates a significant difference.

## 5. Conclusions

In summary, we found that a natural flavonoid inhibited the expression levels of PD-L1 in A549, H292, and H460 NSCLC cells. Nobiletin inhibited tumor progression by regulating STAT3 mediated PD-L1 expression and enhanced the evasion of immunosuppression effects via a p53-independent PD-L1 downregulation. We also found that miR-197 plays a vital role in anti-tumor immunity, as it was negatively co-related with STAT3 and PD-L1 expression. Finally, we also evaluated the role of nobiletin in evasion of immunosuppression through its combination effect with anti-PD-1 mAb in PD1/PD-L1 blockade, which established that nobiletin is a chemotherapeutic drug for immune checkpoint-based therapies. 

## Data Availability

The data presented in this study are available on request from the corresponding author. The data are not publicly available due to personal reasons.

## Supplementary Materials

The following is available online at: www.mdpi.com/article/10.3390/ijms22189843/s1.
