# Supplementary figures and images for: Mechanistic Insights of Anti-Immune Evasion by Nobiletin through Regulating miR-197/STAT3/PD-L1 Signaling in Non-Small Cell Lung Cancer (NSCLC) Cells
Source: Int J Mol Sci. 2021 Sep 11;22(18):9843. doi: 10.3390/ijms22189843 (PMC8468939; doi:10.3390/ijms22189843)

Figure S1

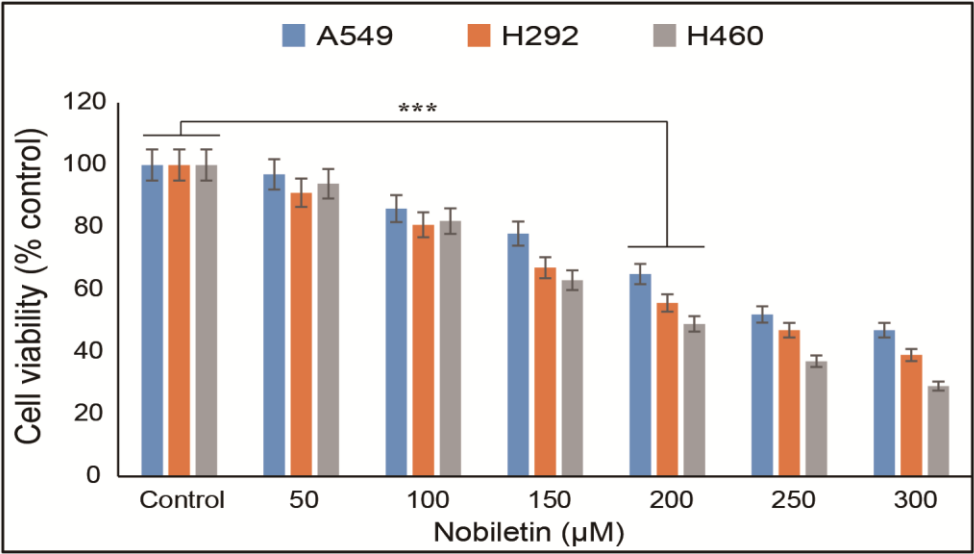

**Figure S2**

**A**

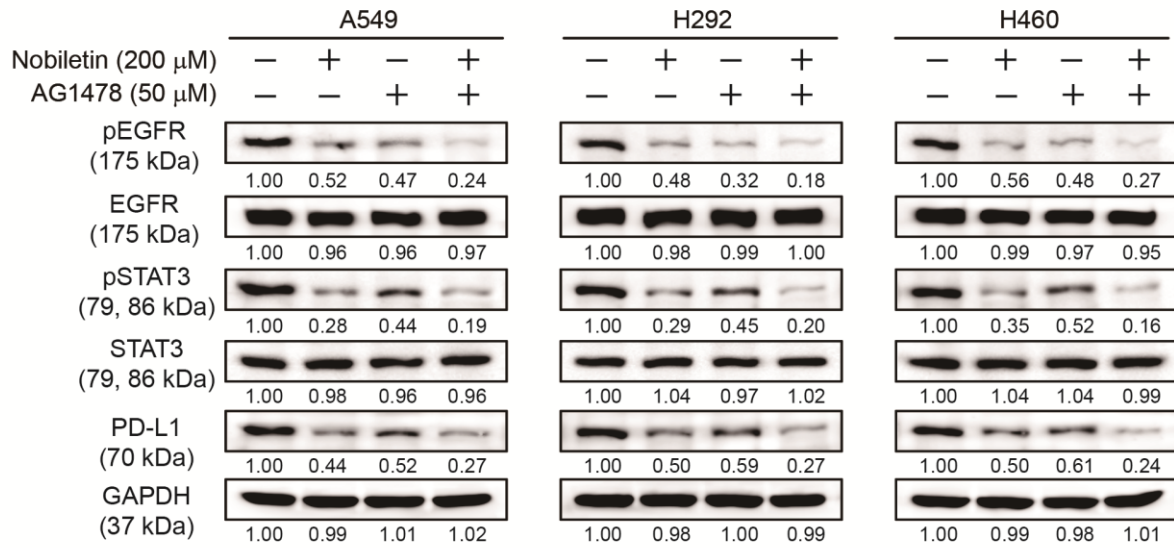

**B**

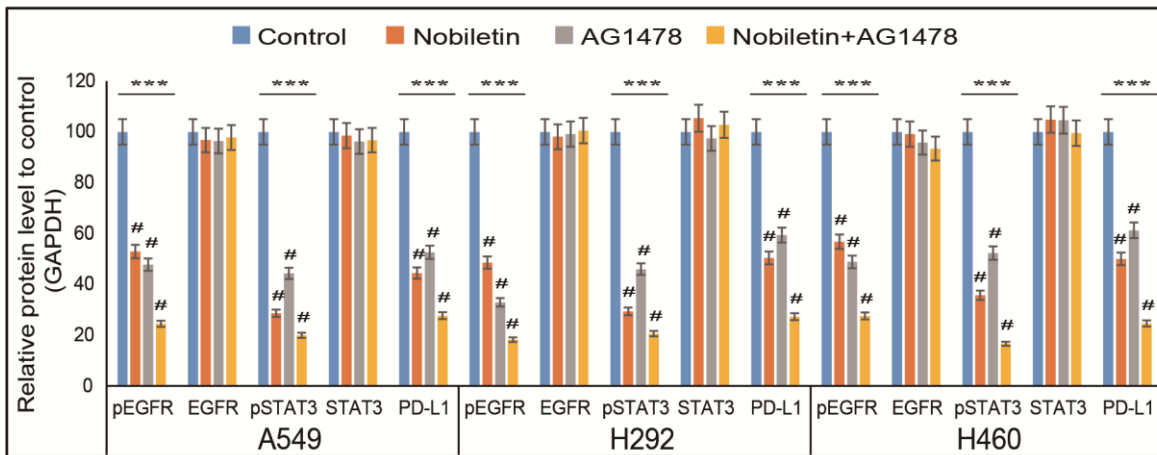

**Figure S3**

**A**

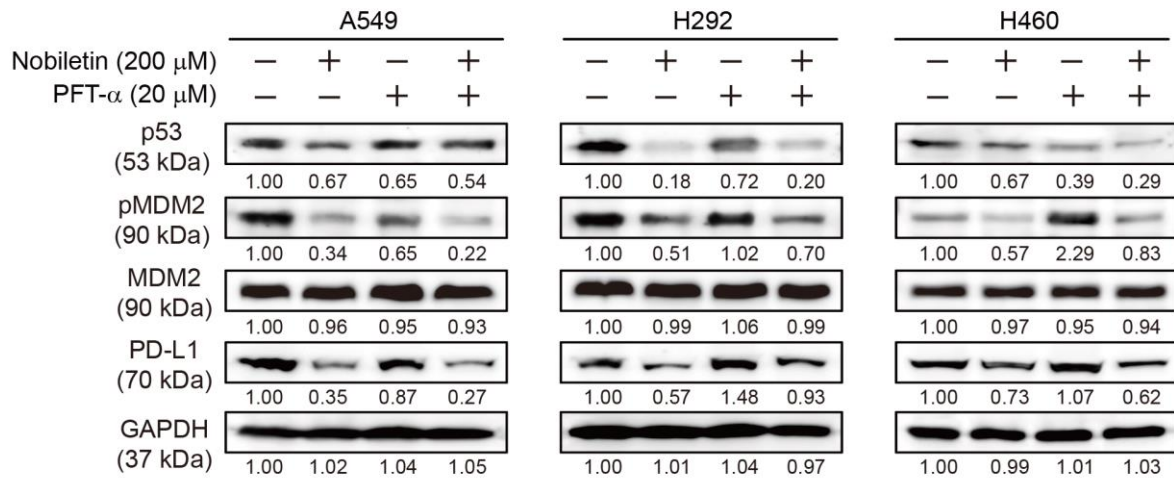

**B**

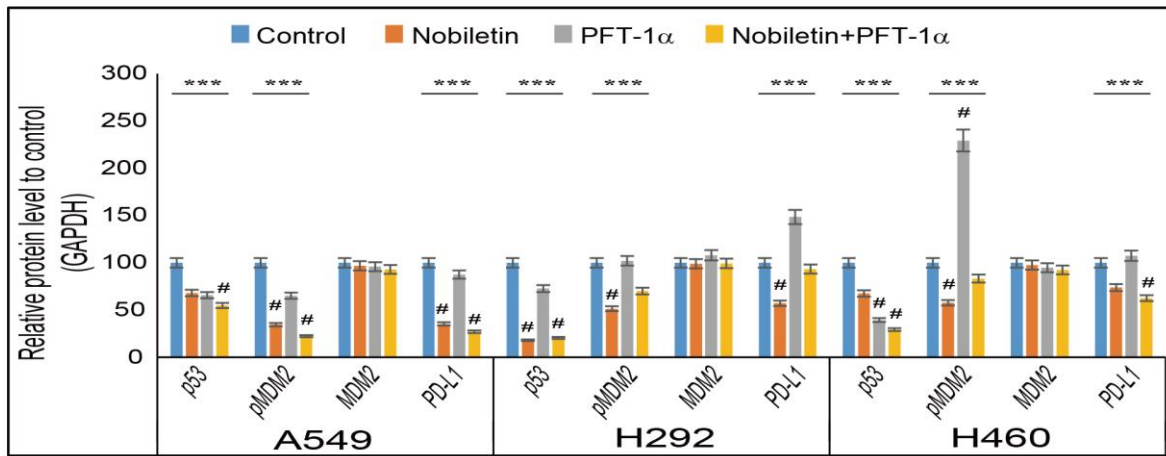

**Figure S4**

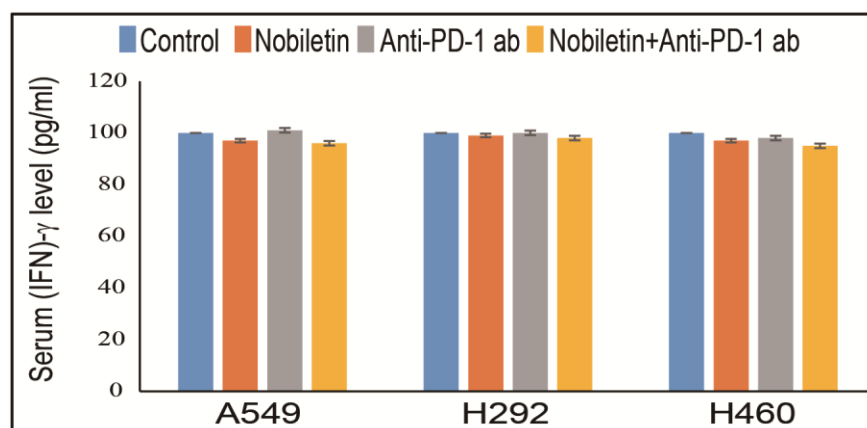

Supplement: Supplementary file 1 [file ijms-22-09843-s001.zip › ijms-1364097-supplementary.pdf]
